# Harnessing the power of theta: natural manipulations of cognitive performance during hippocampal theta-contingent eyeblink conditioning

**DOI:** 10.3389/fnsys.2015.00050

**Published:** 2015-04-13

**Authors:** Loren C. Hoffmann, Joseph J. Cicchese, Stephen D. Berry

**Affiliations:** ^1^Center for Learning and Memory, University of TexasAustin, TX, USA; ^2^Department of Psychology and Center for Neuroscience, Miami UniversityOxford, OH, USA

**Keywords:** neurobiological oscillations, theta, hippocampus, brain-computer interface, cognitive enhancement, local field potential, eyeblink classical conditioning

## Abstract

Neurobiological oscillations are regarded as essential to normal information processing, including coordination and timing of cells and assemblies within structures as well as in long feedback loops of distributed neural systems. The hippocampal theta rhythm is a 3–12 Hz oscillatory potential observed during cognitive processes ranging from spatial navigation to associative learning. The lower range, 3–7 Hz, can occur during immobility and depends upon the integrity of cholinergic forebrain systems. Several studies have shown that the amount of pre-training theta in the rabbit strongly predicts the acquisition rate of classical eyeblink conditioning and that impairment of this system substantially slows the rate of learning. Our lab has used a brain-computer interface (BCI) that delivers eyeblink conditioning trials contingent upon the explicit presence or absence of hippocampal theta. A behavioral benefit of theta-contingent training has been demonstrated in both delay and trace forms of the paradigm with a two- to four-fold increase in learning speed. This behavioral effect is accompanied by enhanced amplitude and synchrony of hippocampal local field potential (LFP)s, multi-unit excitation, and single-unit response patterns that depend on theta state. Additionally, training in the presence of hippocampal theta has led to increases in the salience of tone-induced unit firing patterns in the medial prefrontal cortex, followed by persistent multi-unit activity during the trace interval. In cerebellum, rhythmicity and precise synchrony of stimulus time-locked LFPs with those of hippocampus occur preferentially under the theta condition. Here we review these findings, integrate them into current models of hippocampal-dependent learning and suggest how improvement in our understanding of neurobiological oscillations is critical for theories of medial temporal lobe processes underlying intact and pathological learning.

## Overview

In attempting to characterize the necessary and sufficient properties of electrical information processing in the brain, there have been many candidates for the most useful or prestigious signals to record and analyze. Theodore Holmes Bullock, a pioneering figure in modern neuroscience, made the argument that progress would require systematic observation of neural activity in many bandwidths and at many timescales (Bullock, 1997). Interactions between and among such observations might be more than the sum of their parts. As part of this aggregation of signals, neurobiological oscillations, measured in local field potential (LFP) activity, represent waves of current flow through an extracellular region as a result of the ionic conductance associated with neuronal excitability (Llinás, 1988). There is a well-established systematic relationship between LFPs and neuronal unit activity, in that most units have a preferential phase of a simultaneously occurring oscillation, at which they are most likely to emit action potentials (Green et al., 1960; Buzsáki et al., 1983). Furthermore, neurons can change their preferred phase of an oscillation as a means of coding temporal information during activities such as spatial navigation (O’Keefe and Recce, 1993; Skaggs et al., 1996). Although, the definitive functions and underlying mechanisms of neurobiological oscillations as a whole continue to emerge; the role of rhythmic oscillations as timing signals, organizing and coordinating activity amongst different cells, neural ensembles or regions of the brain is well-established (Singer, 1999; Buzsáki, 2002; Lisman, 2005; Llinás, 2009). Oscillatory potentials in neurobiological systems exist in a range of frequency bands, or cycle lengths, from delta waves (~0.02–3 Hz; 50 s−333 ms cycles) to high frequency gamma (80–200 Hz; 12.5–5 ms cycles) (Buzsáki, 2006). The length of a frequency’s cycle provides a temporal window for encoding and transfer of information that can be precisely repeated within a neural network. Furthermore, each frequency band is associated with a specific set of overt and covert behaviors and can differ in underlying cell assemblies, mechanisms and pharmacological influences to support a given cycle length. Theta band oscillations are one such frequency in the brain that has gained increasing attention in the last few decades as a phenomenon closely associated with states of arousal, attentiveness and active cognition. As sinusoidal waves ranging from 3–12 Hz, theta is within the bandwidth of oscillations that have been proposed to synchronize large areas or across long distances (Green and Arduini, 1954; Buzsáki, 2002; Canolty et al., 2006) but also play a role in local circuit organization and synaptic plasticity (O’Keefe and Recce, 1993; Hyman et al., 2003; Hasselmo, 2005; Klausberger and Somogyi, 2008; Cutsuridis et al., 2010). Though theta can be observed in a variety of brain regions, this frequency has been most extensively studied in the hippocampus due to its early discovery and continued relevance within this region. Early work identified the accompaniment of 3–7 Hz theta synchronization in the hippocampus with states of alert immobility (Winson, 1972). Vinogradova (1995) posited that theta rhythm was a mechanism of selective attention, which could be viewed a prerequisite for memory formation. Furthermore, a growing number of studies evidence the role of theta in hippocampus-dependent tasks, such as encoding and retrieval of declarative memory (Klimesch et al., 1996; Hasselmo, 2005; Osipova et al., 2006), spatial navigation (O’Keefe and Recce, 1993; Skaggs et al., 1996; Kahana et al., 1999; Vertes, 2005; Burgess and O’Keefe, 2011), classical and instrumental conditioning (Berry and Thompson, 1978; Jurado-Parras et al., 2013) and working memory maintenance (Hwang et al., 2005; Jones and Wilson, 2005).

Manipulation of theta is key to exploring these relationships. Often-used pharmacological or lesion methods typically produce permanent or long-lasting theta or non-theta states that can distort relations between theta and, for example, cellular activity (Scarlett et al., 2004). In addition, unlike the 8–12 Hz movement-related non-cholinergic theta that can continue indefinitely as long as an animal is locomoting, the 3–7 Hz theta typically seen during cognitive processes in rabbits, monkeys and humans waxes and wanes throughout a session—thus being difficult to model with lesions or drugs. Using a custom-designed brain-computer interface (BCI) that allows the natural fluctuation of theta states, our lab has established that hippocampal theta reflects a brain state conducive to rapid acquisition of the stimulus contingency underlying eyeblink conditioning. Along with behavioral enhancements, theta-triggered training has consistently demonstrated increases in LFP and multi-unit responses in the hippocampus, modulation of single-unit response patterns, changes in prefrontal cortex multi-unit responses, and rhythmicity in cerebellar LFPs. Our BCI allows us to engage the hippocampal theta rhythm in a manner that may allow precise timing of signals within the hippocampus and across regions necessary for acquisition of a trace conditioning task. Such research on the impact of normal/optimal patterns can underscore the positive aspects of synchrony, while theta disruptions can reveal maladaptive patterns that may correlate with dysfunctional cognitive processes. Here we present our systematic approach to the study of hippocampal theta’s role in learning, including an overview of hippocampal theta origins, our model system for studying theta, and the contributions of theta at the behavioral, LFP, multiple- and single-unit levels. These results provide support for existing models of learning focused on both pharmacological systems and oscillatory mechanisms.

## Septohippocampal Theta

Early reports have identified a 3–7 Hz frequency band as atropine-sensitive, or Type II, theta due to its disruption by the competitive cholinergic inhibitor, atropine, and have distinguished it from a higher, 8–12 Hz frequency band, deemed atropine-resistant, non-cholinergic, or Type I, theta despite technically falling within the alpha bandwidth in most literatures including those classically defined by human scalp EEG (Kramis et al., 1975). These two frequency bands in hippocampal LFPs have been further distinguished on the basis of anatomical and pharmacological substrates, overt and covert behavioral correlates, species differences and common paradigms in which each are studied. Type II theta is disrupted by atropine and other cholinergic antagonists but is insensitive to anesthetics such as urethane, diethyl ether, pentobarbital and alcohol, while Type I theta displays the opposite pattern of affected manipulations. Type II theta is known to rely on cholinergic, GABAergic and glutamatergic projections from the medial septum (MS; Mesulam et al., 1983a,b; Freund, 1989; Leranth and Frotscher, 1989; Kiss et al., 1990; Smythe et al., 1992; Colom et al., 2005). The neurotransmitters and receptors responsible for Type I theta have not been identified conclusively, but involvement of a serotonergic component has been suggested (e.g., Vanderwolf and Baker, 1986; Robertson et al., 1991). Type I theta is associated with voluntary behaviors, such as locomotion, head movements, and rearing as well as REM sleep. This type of theta is commonly studied in rat spatial navigation. In contrast, Type II theta is predominantly studied in rabbits and other more easily restrained animals and has been shown to correspond uniquely to states of alert immobility and stationary behaviors such as grooming and eating (Harper, 1971; Winson, 1972; Kramis et al., 1975). The type II theta commonly observed in the rabbit corresponds more closely to human theta in frequency, behavioral correlates (such as working memory and sensorimotor integration) and epoch duration (Kahana et al., 2001; Lega et al., 2012). Though most species display Type I and II theta, the prevalence of each type may be, in part, ecologically determined (Winson, 1972; Kramis et al., 1975; Robinson, 1980).

A major research endeavor has been to understand the mechanisms controlling hippocampal theta oscillations. There are a number of interacting ascending and descending pathways in addition to local circuit properties that contribute to the pacing and generation of 3–7 Hz theta oscillations in the hippocampus and to the information transferred by these oscillations within and between brain structures. An important distinction to make in neural mechanisms of theta is between LFP generators and pacemakers. Generators are defined as active channels on a membrane. Generators contribute to active and passive current summation to produce what are commonly measured as LFPs. The currents associated with the ionic channels in the neural membranes of cells within the hippocampus represent one such generator. Many experimental manipulations and computational models have indicated at least two, but likely several rhythm-generating mechanisms and various theta current dipoles contribute to hippocampal theta oscillations (Leung, 1984; Bland, 1986; Vanderwolf, 1988; Fox, 1989; Kamondi et al., 1998; Konopacki, 1998; Kocsis et al., 1999; Gillies et al., 2002; Traub et al., 2004; Rotstein et al., 2005). In contrast (though, not mutually exclusive), structures with pacemaker functions serve to set the frequency and synchronize oscillatory potentials of populations of generators (Stewart and Fox, 1990). Among the most critical to hippocampal theta generation, pacing and maintenance are the MS and associated vertical limb of the diagonal band of Broca (vDBB), supramammillary nucleus and associated brainstem nuclei (Woolf and Butcher, 1986; Kirk and McNaughton, 1991; Vertes and Kocsis, 1997; Nowacka et al., 2002), CA3 (Buzsáki et al., 1986; Brankack et al., 1993; Sandler et al., 2015), and entorhinal cortex (Petsche et al., 1962; Alonso and Garcia-Austt, 1987; Stewart and Fox, 1990; Lee et al., 1994).

### Medial Septal Pacing

As the main subcortical source of input to hippocampus, the MS-vDBB has been deemed essential to theta periodicity oscillations since lesions or pharmacological inactivation of the MS-vDBB significantly alter the oscillatory pattern and frequency, and, in many cases, result in complete abolition of hippocampal theta (Green and Arduini, 1954; Petsche et al., 1962; Winson, 1978; Andersen et al., 1979; Givens and Olton, 1990; Stewart and Fox, 1990; Smythe et al., 1992). Because hippocampal Type II theta emerges ~500 ms after the MS transitions to theta (Bland et al., 1999) and MS pacemaking interneuron spikes lead hippocampal theta by ~80 ms when maximally phase locked (Hangya et al., 2009), the MS-vDBB is thought to be the key pacemakerof hippocampal theta oscillations (though the recurrent CA3 system can also generate theta as presented below). MS neurons are known to display phasic unit burst firing and during hippocampal theta oscillations as many as 75% show theta-dependent activity (a number that drops as theta ceases; King et al., 1998; Brazhnik and Fox, 1999).

Cholinergic, GABAergic (γ-aminobutyric acid-releasing) and glutamatergic neurons represent the vast majority of the MS cellular population innervating the hippocampus (Lewis et al., 1967; Köhler et al., 1984; Freund and Antal, 1988; Kiss et al., 1990; Gritti et al., 2006). Labeling studies have identified two distinct fiber types projecting from MS-vDBB to the hippocampus and contributing to theta oscillatory activity (Freund and Antal, 1988; Smythe et al., 1992). GABAergic fibers, which target GABAergic interneurons of the hippocampus, and a set of non-GABAergic fibers (putatively cholinergic) which predominantly innervate basal dendritic regions of pyramidal and granule cells. Many studies have reported on the pharmacologic disruptions to theta with cholinergic antagonists such as scopolamine or atropine, which target muscarinic acetylcholine (ACh) receptors expressed on the dendrites and soma of MS neurons. These data demonstrated that cholinergic inactivations of MS-vDBB reduce the regularity and amplitude of theta oscillations recorded in the hippocampus (Kramis et al., 1975; Powell, 1979; Salvatierra and Berry, 1989; Stewart and Fox, 1990; Asaka et al., 2000; Griffin et al., 2004). Data now suggest however, that the timeline for muscarinic effects of ACh in the hippocampus is too slow to pace even the lowest theta frequency oscillation and therefore, is not a major contributor to theta frequency regulation (Stewart and Fox, 1990). Rather, metabotropic cholinergic inputs likely contribute to the maintenance of theta frequency power (i.e., energy in the theta bandwidth; Vinogradova et al., 1998).

In addition to amplitude-mediating cholinergic input, the hippocampus receives GABAergic input from the septum that contributes to gating, reset and phase precession of hippocampal neuronal activity during theta oscillations as well as frequency regulation of theta oscillations (Köhler et al., 1984; Vinogradova et al., 1998; Cutsuridis and Hasselmo, 2012). Freund et al. have demonstrated the selectivity of MS GABAergic projections, which synapse almost exclusively on GABAergic interneurons of the hippocampus (Freund and Antal, 1988; Kiss et al., 1990). This allows for disinhibition of hippocampal pyramidal cells, playing an important role in rhythmic synchronization. MS microinfusions of the GABA_A_ receptor agonist, muscimol, in urethane-anesthetized rats decreased the amplitude of theta at low concentrations and completely abolished hippocampal theta oscillations at higher concentrations (Bland et al., 1996). GABAergic pacemaker neurons in MS lead hippocampal theta activity (and its interneurons), further supporting its role as a driver of hippocampal theta (Varga et al., 2008). Population activity of GABAergic neurons concentrates around the trough (178 degrees) and peak (330 degrees) of the theta cycle (Borhegyi et al., 2004). These GABAergic and other neuron populations (cholinergic and glutamatergic projections) send pacing inputs to hippocampus via the fimbria-fornix (Amaral and Kurz, 1985; Hajszan et al., 2003; Sotty et al., 2003) which, when transected, results in abolition of hippocampal theta (M’Harzi and Monmaur, 1985).

Moreover, both *in vitro* (Konopacki and Gołebiewski, 1992; Konopacki et al., 1997) and *in vivo* studies (Colom et al., 1991; Smythe et al., 1992; Vinogradova et al., 1998) provide evidence that the GABAergic and cholinergic systems interact to produce the amplitude and frequency components of hippocampal theta rhythm. In addition to their localization on GABAergic neurons, cholinergic neurons of the MS-vDBB are known to express specific GABA_A_-receptor subunit repertoires (rat and marmoset; Gao et al., 1995). Septohippocampal cholinergic neurons are modulated by GABAergic inhibitory inputs via these GABA receptors, which are expressed exclusively on cholinergic neuron cell bodies (Wood et al., 1979; Allen and Crawford, 1984; Moor et al., 1998). Wood et al. (1979) reported significant decreases in cholinergic turnover rates in hippocampus following MS inactivation with muscimol, while muscimol inactivation of MS drastically reduces both acetylcholine utilization and concurrent 6–9 Hz theta oscillations in the hippocampus (theta decreased in both amplitude and rhythmicity; Allen and Crawford, 1984). Stewart and Fox (1990) proposed a model whereby GABAergic and cholinergic septal afferents synapse on the same hippocampal interneurons, and consequently, pace theta field activity. Conversely, it has also been demonstrated more recently that ACh primarily and profoundly excites septohippocampal GABAergic neurons (rather than cholinergic neurons) when muscarinic cholinergic agonists were infused into the MS-vDBB (Wu et al., 2000) and that muscarinic drug effects during mnemonic tasks are mediated predominantly by GABAergic septohippocampal projections rather than the cholinergic pathway (Alreja et al., 2000). Together these findings indicate that the MS-vDBB acts as a pacemaker of hippocampal theta through dynamic interactions between cholinergic and GABAergic projections. MS glutamatergic projections to hippocampus have also been shown to produce rhythmic discharges in the theta range (Huh et al., 2010) but their role in the genesis of the hippocampal theta oscillations is less established. The hippocampo-septal back-projection, composed of GABAergic interneuron inputs from CA1-CA3 stratum oriens that preferentially innervate MS GABAergic cells (Alonso and Köhler, 1982; Tóth et al., 1993) has also proven to be essential for synchronizing the medial septal and hippocampal circuits and possibly amplifying the theta frequency pacing inputs from MS (Tóth et al., 1993; Wang, 2002).

### Brainstem and Hypothalamic Pacing

MS-vDBB is known to receive inputs from the pedunculopontine tegmental nucleus and laterodorsal tegmental nucleus. These cholinergic brainstem nuclei exert influence on MS-vDBB and theta generation both via direct afferents to MS-vDBB (Woolf and Butcher, 1986; Hallanger and Wainer, 1988) and via an indirect route through the supramammillary and posterior hypothalamic nuclei (Kirk and McNaughton, 1991; Oddie et al., 1994). Direct stimulation of pedunculopontine tegmental nucleus and laterodorsal tegmentum elicits cortical desynchronization and hippocampal theta oscillations (Datta and Siwek, 1997). Suppression of the pedunculopontine tegmental activity with local procaine infusion abolishes urethane-resistant theta oscillations in rats (Nowacka et al., 2002). One explanation of pacing dynamics that has been put forth (Kirk, 1998) is that MS firing properties are driven largely by projections from the supramammillary nucleus. In this scenario, supramammillary nucleus and posterior hypothalamic nucleus receive tonic, non-cholinergic inputs from the reticularis pontis oralis (Vertes, 1977; Vertes et al., 1986), which is targeted by cholinergic projections from the pedunculopontine tegmental nucleus (Mitani et al., 1988; Semba et al., 1990). Inputs from both supramammillary nucleus and posterior hypothalamic nucleus, in turn, project to cholinergic and GABAergic MS-vDBB neurons and act in synergy to control its theta-pacing influences on the hippocampus. However, there are reciprocal connections between MS and supramammillary nucleus, and both can generate theta activity (even when separated from each other and/or hippocampus; Kirk and McNaughton, 1991; Oddie et al., 1994; Thinschmidt et al., 1995; Kirk et al., 1996; Kirk, 1998; Denham and Borisyuk, 2000). A likely alternative scenario is one in which independent oscillators of the MS and supramammillary nucleus are dynamically coupled so that the origin of theta can shift between these regions, depending on the type of theta or the animal’s internal state. Additional evidence for this proposal has been presented in a study by Kocsis (2006), which demonstrated that bidirectional coupling between theta generators (MS or supramammillary nucleus) could be led by either one structure or the other in urethane-anesthetized rats. Supramammillary spike trains were consistently predictive of MS theta rhythmicity during episodes of sensory-evoked or brainstem-stimulated theta as well as during acceleration of theta rhythm. In contrast, spontaneous slow theta and the deceleration of theta frequency and corresponding unit firing of a subpopulation of supramammillary neurons were led, and perhaps driven, by descending input from the septohippocampal system.

### Intrinsic Oscillators in Hippocampus

Interestingly, both *in vitro* models and *in vivo* recordings of LFP theta rhythms have shown that circuitry intrinsic to the hippocampus and entorhinal cortex can generate theta oscillations in the absence of extrinsic timing inputs (Kocsis et al., 1999; Traub et al., 2004; Goutagny et al., 2009; Brandon et al., 2011). Theta current dipoles within these structures derive from a number of sources, including voltage-dependent membrane resonance (Leung and Yim, 1986; Kamondi et al., 1998; Leung and Yu, 1998; Hu et al., 2007), burst-induced somatic hyperpolarization (Hu et al., 2007), dendritic calcium spikes (Magee and Johnston, 1995; Schiller et al., 1997) and, at the microcircuit level, synaptic currents generated from a diversity of layer-specific excitatory and inhibitory inputs from external sites as well as intraregional recurrent networks of interneurons and pyramidal cells (Amaral and Witter, 1995; Freund and Buzsáki, 1996; Gillies et al., 2002; Klausberger et al., 2003; Goutagny et al., 2009; Buzsáki and Wang, 2012). The variety of mechanisms involved suggests that a large degree of computational flexibility is possible through varying the frequency and phase of individual dipoles.

Within the hippocampus, theta current oscillations arise from the multiple layers of densely-packed pyramidal and granule cells that constitute the CA3-CA1-subicular layer and dentate gyrus, with rhythmic excitatory input from entorhinal cortex through the perforant path to distal apical dendrites of CA1 and CA3 pyramidal cells as the major current generator (Montgomery et al., 2009). Entorhinal input also targets dentate gyrus granule cells, whose rhythmic activity is, in turn, conveyed through mossy fibers to CA3 pyramidal cells and then, through the Schaffer collaterals, to CA1 pyramidal cells (Kamondi et al., 1998). It is this type of organization and segregated inputs to the hippocampus that allows for the emergence and the coexistence of multiple phase-shifted current dipoles during theta. Current-source density analysis using multisite recordings of the laminar profile of CA1 LFPs has facilitated the assessment of the distribution of the synaptic currents involved in hippocampal theta generation while eliminating passively conducted signals (Mitzdorf, 1985). Indeed, it has been shown that theta oscillations exhibit a relatively continuous phase shift across the different anatomical layers in the dorsal-ventral axis of the hippocampus from CA1 stratum pyramidal to dentate gyrus (Lee et al., 1994; Bragin et al., 1995; Kamondi et al., 1998). Since it would be expected that a single current source would display an abrupt 180-degree phase shift, this observation supports the existence of more than one current generator. This point is further honed by observations that entorhinal cortex lesions and systemic injection of NMDA blockers both reduce theta oscillations only in selected layers of the hippocampus (Kamondi et al., 1998). It is now clear that the majority of hippocampal layers generate one or more theta current dipoles that are somewhat independent from the global theta rhythm (Kocsis et al., 1999; Montgomery et al., 2009). Such independence among dipoles is due, in part, to activation of a number of inhibitory interneuron subpopulations with similar axonal projections by excitatory inputs of varying origins. Activating these interneurons leads to the generation of local outward theta currents by layer-specific inhibitory dipoles. These local currents reinforce or compete with the inward-directed excitatory currents, depending on their phase relationship (Buzsáki, 2002).

While CA1 and dentate gyrus are endowed with the current generating capabilities described above, CA3, which forms an auto-associative network through its excitatory recurrent collaterals, also has rhythm generating capabilities that contribute directly to theta field recordings in CA3 and CA1 (Buzsáki et al., 1986; Brankack et al., 1993; Sandler et al., 2015). The CA3 intrinsic theta pacemaker functions relatively independently of other theta generators, with frequency and phase changes that vary from extrahippocampal theta inputs (Kocsis et al., 1999). Although this oscillator does not require external timing inputs, it does require a permissive MS-vDBB cholinergic activation since, the theta remaining in the absence of entorhinal input is abolished by anticholinergics (Kramis et al., 1975; Vanderwolf and Leung, 1983; Amaral and Witter, 1989; Kocsis et al., 1999).

## Eyeblink Conditioning as a Model System

Eyeblink conditioning (EBC) is a common form of associative learning used widely in human (Daum et al., 1989; Clark and Squire, 1998) and non-human animals (Gormezano et al., 1962). All forms of EBC involve, at minimum, the presentation of a neutral conditioned stimulus (CS) such as a tone, whisker stimulation or a flash of light, and an unconditioned stimulus (US), such as a corneal airpuff or periorbital stimulation, which consistently evokes some type of reflexive, unconditioned response (UR) in the animal. Over repeated CS-US pairings, the animal learns to associate the two stimuli and emit an adaptive, anticipatory conditioned response (CR) prior to the reflexive UR. For biological analysis, EBC provides an important advantage over complex forms of learning in that the stimuli are well-defined and can be precisely controlled. Due to its well-mapped circuitry and application to humans, this type of discrete sensorimotor learning serves as an ideal animal model for studying the role of oscillatory state on basic learning and timing processes, distributed network interactions and various types of memory with interspecies correspondence.

**Figure 1:**
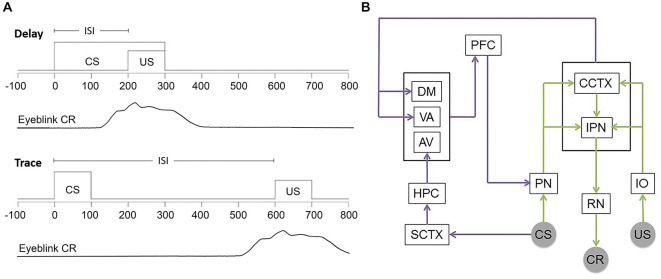
**Delay and Trace EBC training and involved circuitry. (A)** Commonly used temporal parameters for EBC stimuli. Time = 0 ms indicates trial onset. Behavioral learning is measured by the number of CRs emitted by the subject. Adaptive eyeblink closures begin prior to US onset and extend into the US period in order to avoid the aversive stimulus. **(B)** Simplified schematic showing the circuitry essential for both delay and trace (green and purple) EBC and the forebrain circuitry critical to trace EBC. US information entering via trigeminal nucleus projects from the inferior olive (IO) to the cerebellar cortex (CCTX) and interpositus nucleus (IPN) via climbing fibers. CS-related information projects from auditory nuclei (cochlear nucleus and medial auditory thalamic nuclei) to the pontine nuclei (PN) to the CCTX and IPN through mossy fibers. CR performance is controlled by the IPN via the red nucleus (RN) and motor nuclei. The hippocampus (HPC) receives CS information through the sensory cortex (SCTX) and projects to the prefrontal cortex (PFC) through the anterior-ventral (AV) nucleus of the thalamus. Additionally, PFC receives input from the cerebellum through the dorsomedial (DM) and ventral-anterior (VA) nuclei. Persistent trace activity from the PFC is projected to the PN allowing for performance of the trace paradigm.

A number of variations of this task have been developed. Delay and trace EBC (Figure 1A) represent the most common forms and are importantly distinguished both by the duration of the inter-stimulus interval between CS onset and US onset as well as the neural substrates required in each case (Smith et al., 1969). In the simplest form of the task, delay EBC, the CS overlaps and co-terminates with the US leading to a learning framework based on contiguity of stimuli. Inter-stimulus intervals between 200–700 ms are typically used in rabbit preparations. In contrast, trace EBC involves a CS that ends prior to US delivery. This results in a stimulus-free “trace” interval separating the two stimuli. Inter-stimulus intervals typically range from 600–1000 ms in the rabbit and require a higher form of contingency learning by the subjects. This imposes additional demands on the subject as the CS and US do not co-occur and information about the CS must therefore be retained or “remembered” by the involved neural circuitry over a period of time between the stimuli.

All forms of EBC rely critically on the cerebellum and related brainstem circuitry (delay: McCormick et al., 1982; trace: Woodruff-Pak et al., 1985; for reviews see Attwell et al., 2002; Thompson, 2005; in humans see, Gerwig et al., 2007; Cheng et al., 2008; See Figure 1B for basic circuitry schematic). The convergence or close temporal proximity of the CS and US information within the cerebellum is thought to drive learning (Kalmbach et al., 2009; Weiss and Disterhoft, 2011; Siegel et al., 2012). For both delay and trace preparations, lesions restricted to the anterior interpositus nucleus (IPN) completely and permanently prevent acquisition of CRs in naïve animals and abolish CRs in well-trained animals without affecting the reflexive, UR performance (Lincoln et al., 1982; McCormick et al., 1982; McCormick and Thompson, 1984; Yeo et al., 1985a). Data have also shown that regions of the cerebellar cortex, namely anterior cortex and Larsell’s hemispheric lobule VI (HVI), are critical for delay EBC (Garcia et al., 1999; Attwell et al., 2001; Kishimoto et al., 2002) and play at least a modulatory role in precise timing, amplitude and expression of the CR in trace and other more complex EBC preparations (Woodruff-Pak et al., 1985; Berthier and Moore, 1986). Some data argue that the role of cerebellar cortex is the same in delay and trace preparations (Yeo et al., 1985b; Attwell et al., 1999; Kalmbach et al., 2010).

Following termination of sensory input, unit recordings have revealed that no residual activity persists in the cerebellum for longer than ~300 ms (Ito, 1982). As opposed to delay EBC stimuli timelines, it is therefore unlikely that the cerebellum is able to independently maintain a CS representation through the duration of the trace interval for subsequent convergence of CS-US information. Thus, in addition to cerebellar-brainstem, trace EBC appears to require the hippocampus (Solomon et al., 1986; Moyer et al., 1990; Weiss et al., 1999) and other forebrain areas such as the medial prefrontal cortex (mPFC) when the stimulus-free period is long enough (500-ms for rabbits; Woodruff-Pak et al., 1985; Kronforst-Collins and Disterhoft, 1998; Weible et al., 2000; Takehara et al., 2003). Most current interpretations of hippocampal function in trace EBC center around forebrain involvement in sustaining CS-related activity through the trace period until US onset early in training (Clark and Squire, 1998; Woodruff-Pak and Disterhoft, 2008; Kalmbach et al., 2009; Weiss and Disterhoft, 2011; Siegel et al., 2012; Siegel and Mauk, 2013; Suter et al., 2013). However, current thinking on this idea hinges upon direct connections between mPFC and pontine nuclei as well as hippocampal modulation of mPFC through indirect means, since no direct connections between hippocampus and pons, cerebellum or mPFC have been identified in the rabbit. The specific interactions between hippocampus and mPFC or hippocampus and cerebellum that may underlie forebrain involvement in trace EBC have yet to be precisely identified.

A growing body of data suggests bidirectional influences between hippocampus and cerebellum. Lesion or inactivation of the cerebellum disrupts hippocampal learning-related unit responses during both delay and trace, though the underlying mechanisms of this outcome have not been determined (delay: Clark et al., 1984; Ryou et al., 1998; trace: Sears and Steinmetz, 1990). It is therefore thought that the hippocampus depends on conditioning-related inputs from IPN and/or red nucleus for neural CR activity to be exhibited (Ryou et al., 1998). Converging lines of evidence leave open the possibility that the hippocampus exerts influence on cerebellum as well. Hippocampal lesions and disruption of the septohippocampal system abolish trace EBC (Berry and Thompson, 1979; Solomon et al., 1983; Salvatierra and Berry, 1989; Kaneko and Thompson, 1997; Takehara et al., 2003). Further, direct connections between cerebellar nuclei/folia and hippocampus have been demonstrated in cats, rats, monkeys and humans, (Heath and Harper, 1974; Snider and Maiti, 1976; Heath et al., 1978; Newman and Reza, 1979; Liu et al., 2012; Arrigo et al., 2014). In one of the first studies to compare unit recordings in hippocampus and cerebellum during EBC, Green and Arenos (2007) recorded from each region during both trace and delay in rats and characterized unit responses in these areas during CR vs. non-CR trials. These data showed a shift in IPN activity from early to later in the trace period over the course of trace EBC, which the authors suggested may reflect the influence of hippocampus (through PFC/pontine nuclei) on the cerebellar unit responses. Since acquisition of EBC critically involves the cerebellum, some type of alteration in cerebellar activity would be expected.

Eyeblink conditioning in humans appears to follow the same general laws and depends upon similar neural circuit interactions to those reported in the rabbit literature (Steinmetz and Woodruff-Pak, 2000). Patients with cerebellar damage are impaired in EBC and display none of the motor deficits that would be expected to disrupt unconditioned eyeblink responses (Solomon et al., 1989; Daum et al., 1993; Topka et al., 1993; Woodruff-Pak et al., 1996). Additionally, patients with hippocampal damage via medial temporal lobectomy can successfully learn delay EBC, but fail to learn more complex paradigms, such as trace eyeblink (Daum et al., 1989, 1991; Gabrieli et al., 1995; McGlinchey-Berroth et al., 1997; Clark and Squire, 1998). Involvement of the prefrontal cortex has also been extended from animal models to human studies with corroborative results (Blaxton et al., 1996; Schreurs et al., 1997).

Due to extensive and systematic mapping of the critical neural substrates, this paradigm is among the most well-understood and widely used neurobiological model for mammalian associative learning. Such work in animal models of EBC have demonstrated close behavioral parallels with human EBC, lending support to the hypothesis that the learning shares common neurobiological substrates in humans and other mammals. These factors place rabbit EBC in an ideal position for use as a model system to study the roles and potential mechanisms of less understood phenomena (such as neurobiological oscillations) with a high level of validity and applicability to human populations.

## Development of the BCI Methodology

The ebb and flow of theta is a common feature across species, presumably allowing for its well-documented role in transient and state-specific timing functions (Green and Arduini, 1954; Kramis et al., 1975; Buzsáki, 2005; Lega et al., 2012). Typically, rabbits show episodes of theta that last for a few seconds, interspersed with irregular activity composed of several frequencies above and below the theta bandwidth. Delta (0.5–2.5 Hz), alpha (8–12 Hz), beta (12–30 Hz), low gamma (30–90 Hz), high gamma (>90 Hz) and ripple (150–250 Hz) have been reported to occur in the awake rabbit. Development of a computerized neurophysiological interface that exploited the natural fluctuations in oscillatory state derived from the discovery that amount of spontaneous hippocampal theta present prior to delay EBC was correlated with a faster learning rate in rabbits (Berry and Thompson, 1978). Using 2-min pre-training LFP recordings from hippocampal CA1 region of rabbits prior to delay EBC, a zero-crossing analysis of LFPs showed that the amount of time characterized by a high proportion of 2–8 Hz compared to 8–22 Hz activity correlated negatively with the number of trials required to reach behavioral learning criterion. Thus, more theta yields faster learning. Additionally, Berry (1982) demonstrated a significant correlation between the change in the amount of hippocampal theta activity across training and learning rate, with fast learners moving towards a less synchronized state and slow learners towards a more synchronized theta state. This resulted in a higher similarity in the amounts of theta activity in all subjects after conditioning. These findings stressed the value of using extracellular LFPs as an index of neural processes, specifically hippocampal state, conducive to synaptic modification for learning. More recently, these original results have been replicated (Nokia et al., 2008) and extended to human preparations (Caplan et al., 2001, 2003).

It has since been well documented that treatments disrupting hippocampal theta impair the acquisition of EBC (Berry and Thompson, 1979; Solomon and Gottfried, 1981; Solomon et al., 1983; Salvatierra and Berry, 1989; Solomon et al., 1993; Kaneko and Thompson, 1997; Fontán-Lozano et al., 2005), while studies artificially eliciting or enhancing theta accelerate behavioral learning (Landfield and Lynch, 1977; Wetzel et al., 1977; Deupree et al., 1982; Berry and Swain, 1989; Huerta and Lisman, 1993; Kirov et al., 2009; Tsanov and Manahan-Vaughan, 2009). One drawback to such lesion and drug studies is that they produce unnatural brain states, due to their permanent modification of the systems involved in theta and their inability to specifically coordinate theta with individual conditioning trials (Solomon et al., 1983). In a conditioning session, rabbit hippocampal theta typically occurs in epochs varying from 2 cycles to several seconds in duration, interrupted by periods of non-theta (either large irregular activity or sharp waves). This natural ebb and flow may be an important aspect of theta in cognitive processes (Buzsáki, 2002). Long-lasting or permanent treatments such as drugs or lesions prevent this natural fluctuation and may induce LFPs that look like theta but interact differently with the endogenous neural substrates. For example, electrical stimulation of the MS (producing theta field potentials in hippocampus) has been shown to produce aberrant, “non-physiological” activity patterns in theta-related hippocampal cells (Scarlett et al., 2004). One solution to this important problem of maximizing theta would be to let natural variation occur but restrict trials to make sure they coincide with endogenous hippocampal theta, as pursued in studies from our lab.

**Figure 2:**
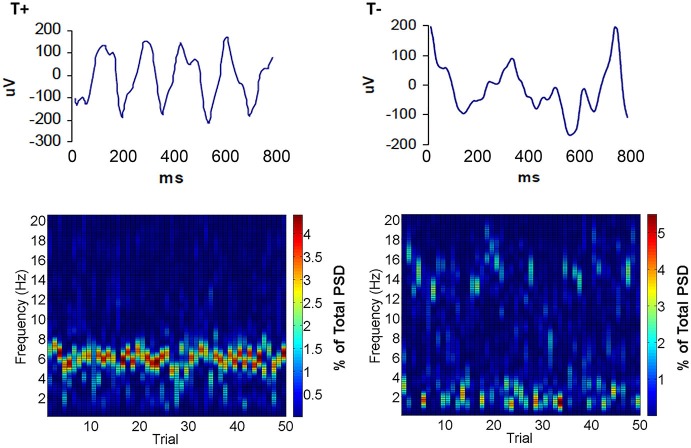
**Examples of hippocampal LFPs that triggered trials in the T+ and T− groups**. Notice that local field potential (LFP) activity during T+ trials (top left) showed a predominance of activity in the theta band. Conversely, hippocampal activity during T− trials (top right) showed a mixture of frequencies higher and lower than the theta band. Below are surface plots of spectral density illustrating typical distributions of frequencies within a 0–20 Hz bandwidth that triggered each training trial in a daily session for T+ (bottom left) or T− conditions (bottom right). Note that T+ spectrograms have high theta and low delta and alpha, while T−, in this case is characterized by low theta and high delta or alpha across trials.

In our attempt to discern the significance of naturally occurring theta within conditioning sessions, we developed a BCI to limit eyeblink training to two naturally occurring extremes of hippocampal oscillatory state such that each of two groups can be trained in either the explicit presence (T+) or absence (T−) of on-going theta (Seager et al., 2002). Our methods included chronic implantation of monopolar electrodes located in specific cell layers of hippocampus by recording of electrophysiological patterns during the lowering of electrodes in animals under general anesthesia. Custom bioamplifiers provide a gain of 3500–8000 for bandwidths of 0–25 Hz for LFPs and 500–5000 for unit action potentials. During training, commercial software (LabVIEW) samples LFP data at 100 Hz in the 0–25 Hz bandwidth. Fast Fourier-based power spectral analyses calculate a ratio of theta (3.5–8.5 Hz) to non-theta (0.5–3.5 Hz plus 8.5–12 Hz) during a 640 ms sample of spontaneous LFP data. This ratio was updated every 160 ms with a partially overlapping sample (deleting the first 160 ms of the prior sample and adding the most recent 160 ms). If the ratio was 1.0 or greater for 3 successive samples (960 ms total), a training trial was initiated in the T+ group. If the ratio was below 0.3 for 3 successive samples, then a trial was initiated for the T− group. Thus, the behavioral training groups had trials under opposite extremes of theta in the spontaneous, pre-trial theta LFP (Figure 2). Unlike the pre-session baseline assessed in the 1978 study by Berry and Thompson, these methods created an on-line amplitude measure to restrict trial presentation dependent on frequency components of the hippocampal LFP. Furthermore, it permitted assessment with trial-by-trial “resolution” of how neurobiological oscillations (theta) influence learning rate. This technology served to hold hippocampal theta constant during training trials in the manner of a brain state “clamp” that can either maximize or minimize the impact of naturally-occurring theta states. Rather than forcefully holding this parameter constant, our approach allows natural fluctuations while ensuring that training trials and neural recordings are clearly in one theta state for each treatment group.

**Figure 3:**
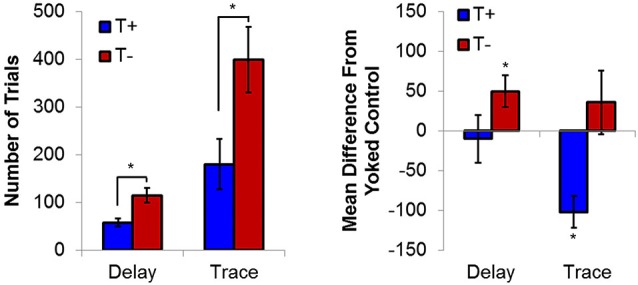
**(Left) Average trials to reach asymptotic responding (8/9 CRs) during delay and trace conditioning**. (Right) Average difference from yoked controls in number of trials to reach asymptote. Hippocampal non-theta contingent trial presentation leads to significant delays in trials to asymptotic responding in delay eyeblink. In contrast, theta-contingent trial presentation significantly accelerates learning in trace eyeblink conditioning. Yoked controls indicate that behavioral effects of theta triggering during delay conditioning are due to detriments in the T− condition, while effects during trace conditioning are due to T+ enhancements. Asterisks indicate *p* < 0.05.

Using this BCI technology, presentation of delay EBC trial presentations during naturally occurring theta in stratum oriens or pyramidale led to significant increases in learning rate relative non-theta conditions (Seager et al., 2002). Animals receiving trials in the explicit presence of theta (T+) reduced the number of trials required to reach asymptotic criterion (8 consecutive CRs out of 9 trials; 8/9 CRs) by nearly half of what was required by animals receiving trials in the explicit absence (T−) of theta (Figure 3). Yoked control groups, in which the inter-trial intervals were matched to T+ or T− subjects but theta was not specifically controlled, allowed for some inferences to be made regarding the direction of the behavioral effect, namely that T− animals performed significantly worse than animals in which theta was unregulated, while T+ animals were not significantly different than either group of yoked controls. These findings related hippocampal function to the rate of delay EBC, supporting previous work showing that the hippocampus, while not essential, plays a modulatory role in the learning of a delay conditioning paradigm (Berry and Thompson, 1978). This is in accordance with several studies concluding that a dysfunctional hippocampus is more detrimental to delay conditioning than complete hippocampal removal(Berry and Thompson, 1979; Solomon and Gottfried, 1981; Salvatierra and Berry, 1989; Stewart and Fox, 1990; Allen et al., 2002; Asaka et al., 2002).

## Theta-Contingent Trace EBC: Learning Enhancements and Hippocampal Modulation

**Figure 4:**
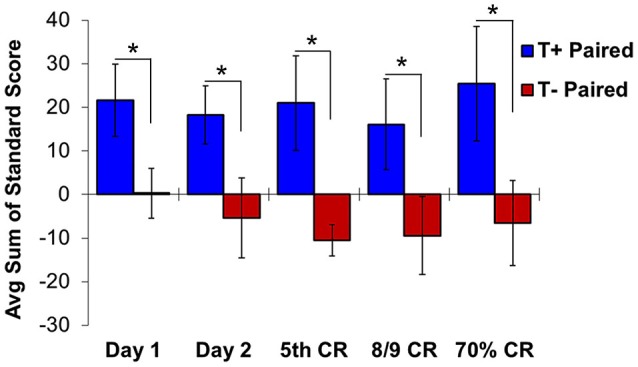
**Hippocampal multiple unit responses during T+ and T− triggered trace eyeblink conditioning show significant differences in average sum of unit standard scores during the second half of the trace interval**. Beginning on day 1 of training, standard scores are significantly higher in T+ than in T− responses. This significant difference continues from early learning (day 1, day 2 and day of 5th CR) through late learning criteria (day of 8/9 CRs and 70% CRs). Asterisks indicate *p* < 0.05.

Given that the hippocampus becomes essential only when the EBC paradigm includes no overlap between CS and US, subsequent studies used the trace paradigm to examine the role of theta in hippocampal-dependent behavioral learning. Unsurprisingly, theta-contingent trace EBC yielded a significantly more robust effect on cognitive enhancement compared to delay manipulations. Animals trained in the T+ condition required significantly fewer trials than T− animals to reach both early (5th CR) and late (8/9 CRs) behavioral criterion (Griffin et al., 2004). The behavioral benefit of theta during early learning was approximately 2.5 times greater for trace EBC than for delay. Comparisons to yoked controls showed that, unlike in the delay paradigm, T+ animals learned significantly faster than their yoked control counterparts in the early and late criteria, while T− animals learned significantly slower than their yoked controls in the early, but not late, criterion. Additionally, hippocampal CA1 multi-unit responses during conditioning generated opposite theta-contingent response patterns during the tone and trace intervals (Figure 4). Beginning on the second day of T+ training hippocampal units showed increased excitation during the CS and trace intervals, while units in the T− groups were inhibited to below baseline levels. This strongly suggests that T− neurons are not simply failing to increase their excitation, but are actively inhibited, consistent with several single-unit response profiles that have been shown during EBC (McEchron and Disterhoft, 1999).

A subsequent study found that CA1 multi-unit responses in T+ animals were significantly more excited during the second half of the trace period than those in the T− condition (Darling et al., 2011)—a difference that was present on the first day of training and continued through the early and late learning phases. As pyramidal cells outnumber interneurons by a large margin in CA1, it is probable that these multi-unit responses strongly reflect hippocampal output. If true, this would suggest that the presence of pre-trial theta allows for persistent excitation through the trace period to downstream structures, such as mPFC, that are essential for bridging the temporal gap between stimuli in trace EBC and perhaps other working memory tasks (Solomon et al., 1986; Takehara-Nishiuchi and McNaughton, 2008; Kalmbach et al., 2009, 2010, 2011; Weiss and Disterhoft, 2011; Siegel et al., 2012).

In addition to the theta-contingent modulation of multi-unit response magnitude, continuing research has demonstrated increased rhythmicity in the theta band of both multi-unit responses (Darling et al., 2011) and hippocampal LFPs (Hoffmann and Berry, 2009; Darling et al., 2011). On the day that CRs began to emerge, autocorrelations revealed that units in T+ paired animals fired at approximately 6.25 Hz during the trace interval, while units in T− animals demonstrated no obvious periodicity during this time window (Darling et al., 2011). The rhythmic firing and enhanced excitation of units in T+ animals is consistent with the interpretation that control of pre-trial theta state allows for increased hippocampal plasticity early in trace EBC. Furthermore, Darling revealed a phase reset of the theta rhythm following CS presentation as well as enhanced amplitude and coherent rhythmicity in T+ animals. This phase reset is similar to what has been shown in rats during working memory tasks, a phenomenon that results in ideal conditions for LTP induction (Givens, 1996; McCartney et al., 2004).

**Figure 5:**
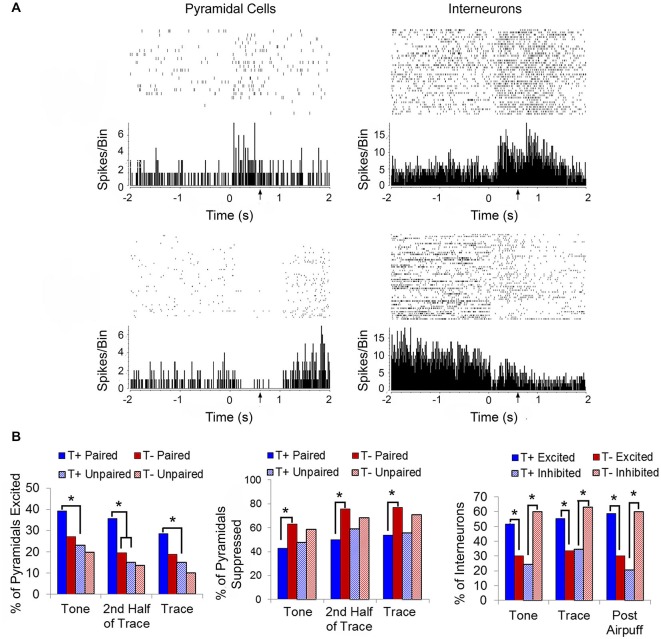
**(A)** Representative raster plots and perievent histograms (10-ms bins) for a pyramidal cell demonstrating an excitatory response to the conditioning stimuli (top left) and a different pyramidal cell in demonstrating a suppressed response to the conditioning stimuli (bottom left). Representative response profile of an interneuron demonstrating excitation to the conditioning stimuli (top right) and a different interneuron demonstrating suppression to the conditioning stimuli (bottom right). **(B)** T+ paired animals had a higher percentage of excited pyramidal cells (left) during the 100-ms tone and 500-ms trace periods than in the T+ unpaired training. When considering only the last half of the trace period, the T+ paired group had significantly more excited pyramidal cells that fired persistently throughout the trace period compared to T− paired animals. T− paired animals showed a greater percentage of suppressed pyramidal cells (middle) during the tone, trace, and 2nd half of trace periods than T+ paired animals. Interneuron responses (right) during the tone, trace, and 1000-ms post airpuff periods were theta modulated with T+ paired animals exhibiting more excited than suppressed interneuron responses compared to T− paired animals, while T− paired animals were more likely to demonstrate suppressed interneuron responses than excited compared to T+ paired animals. Asterisks indicate *p* < 0.05.

While the above studies used chronic electrodes, Darling (2008) implemented tetrode recordings into our BCI studies to extend our theta-modulated electrophysiological findings to hippocampal responses at the identified single unit level. Isolated single neurons were classified as putative pyramidal cells or interneurons based on their firing properties, including waveform duration and spike rate (Fox and Ranck, 1981). Each responsive unit was identified as being excited or suppressed (defined as whether the firing rate was > ±2 standard deviations from the baseline rate during the tone and trace periods, Figure 5A). Such qualitative sorting is increasingly common in analyses of hippocampal unit responses in both EBC studies and place cell firing (Weiss et al., 1996; Brandon et al., 2014; Hattori et al., 2014). During tone presentation, the responses of both interneurons and pyramidal cells were linked to pre-trial theta state. A significantly higher percentage of interneurons in the T+ paired condition were excited than in the T− condition, while a higher percentage of T− paired interneurons were inhibited than in the T+ condition (Figure 5B). This double dissociation of the response pattern serves as a powerful demonstration of the importance of theta in understanding how hippocampal interneurons respond to the CS. Likewise, pyramidal cells in the T− paired condition were more likely to inhibit their firing than those in the T+ condition. Importantly, pyramidal cells in the T+ paired condition were not more likely to be excited than those in the T− paired condition, though they did show a higher likelihood of excitation than cells in the T+ unpaired condition. This suggests that the probability of pyramidal cell excitation was more closely linked to behavioral training than hippocampal theta state during the tone period. This paired/unpaired distinction is consistent with a recent demonstration of prolonged strengthening in the CA3-CA1 circuit during trace conditioning that was not seen in pseudoconditioned animals (Carretero-Guillén et al., 2013). Response patterns during the 500-ms trace period were similar to those seen during the tone presentation. Interneurons in the T+ condition were more likely to be excited than those in T− animals, and T− interneurons were more likely to be suppressed than those in the T+ condition. T− paired pyramidal cells were more likely to be inhibited than T+ paired cells, while T+ paired cells were more likely to be excited than T+ unpaired, but not T− paired cells. Importantly, analysis focused on the second half of the trace period, when adaptive CRs were most likely to be performed, showed that pyramidal cells in the T+ condition were much more likely to be excited than pyramidal cells in T− animals. These late-trace firing differences support an important theoretical distinction between trace vs. delay EBC given that current trace EBC models include a critical role for forebrain structures, such as the hippocampus, in the persistent trace-period unit responses that are within the plasticity intervals necessary in the essential cerebellar EBC circuits (Christian and Thompson, 2003; Siegel et al., 2012).

Since multi-unit recordings reflect the collective responses of neurons in an electrode’s vicinity and the hippocampus exhibits sparse coding (Waydo et al., 2006; Karlsson and Frank, 2008), Darling argued that it is more informative to know which units respond a certain way during EBC rather than how strongly each of those units responds. The range of standard score values in our unit data suggested that the differences in multi-unit responses during theta-triggered EBC might be attributed to a recruitment of more units rather than a large increase in rate for any particular unit. For example, it has been demonstrated that activity-dependent inhibition of pyramidal cells by interneuron basket cells is not due to an increase in the number of action potentials generated by basket cells, but is likely due to the recruitment of additional basket cells or other inhibitory interneurons targeting the same neurons (Sik et al., 1995). Using similar logic, greater excitation may be hypothesized as due to a greater number of excited pyramidal cells in our studies, rather than extreme activation of a few. Although there are large increases in both multi-unit activity and of single pyramidal cell rates when theta is not used to trigger rabbit EBC trials (Berger et al., 1976, 1983; Weiss et al., 1996; McEchron and Disterhoft, 1997, 1999), the effect of theta on the single-unit code in CA1 may be more subtle than the large magnitude standard scores typically reported. That is, the effect of theta may be to change the relative proportion of excitatory pyramidal cell/interneuron recruitment in response to the conditioning stimuli, ultimately guiding adaptive behavior. In the absence of theta, this proportion is non-optimal, resulting in more inhibitory responses in cells of all types and impaired learning. Our findings suggest that the presence of pre-trial hippocampal theta may optimize the magnitude and proportion of excitatory and inhibitory pyramidal cell/interneuron response profiles in a manner that coordinates the neural responses throughout the distributed network supporting trace EBC. The clear differentiation of hippocampal CA1 neural responses along a theta/nontheta dimension has important implications for the impact of theta on the cellular basis of all forms of hippocampus-dependent learning. It would be interesting to extend these studies into additional hippocampal subregions, as research has shown differential alterations in synaptic strength of intrinsic (e.g., CA3-CA1, DG-CA3) hippocampal circuits in relation to learning phase (Gruart et al., 2014).

## Extra-Hippocampal Effects of Theta-Contingent Training

Several theories of hippocampal function have proposed that rhythmic oscillations, such as theta, serve a dynamical coordinating role during learning and other cognitive processes (Singer, 1999; Buzsáki and Draguhn, 2004; Lisman, 2005). As such, it is important to consider the likelihood of hippocampal theta influencing plasticity in other structures essential to trace EBC. To address this issue our laboratory has extended our studies to include recordings from the medial prefrontal cortex (mPFC) and cerebellum during hippocampal theta-contingent training.

**Figure 6:**
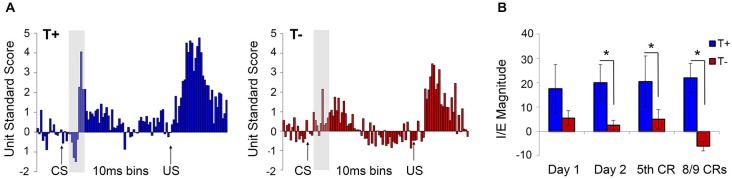
**(A)** Average standard scores for medial prefrontal cortex multiple unit responses during hippocampal theta-contingent trace eyeblink conditioning. As illustrated in the gray highlighted bar following CS onset, T+ animals (blue) had a significantly higher CS-evoked I/E difference scores compared to T− animals (red; *p* < 0.05). T+ prefrontal units also showed significantly greater excitation during the trace (*p* < 0.05) and US periods (*p* < 0.05) relative to the T− units. **(B)** T+ animals showed greater I/E magnitude on day 2 of training that continued through early (5th CR) and late (8/9 CRs) behavioral criterion. Asterisks indicate *p* < 0.05.

The hippocampal-neocortical network represents an important conduit for information flow during learning and memory processes. Hippocampal theta is known to selectively modulate PFC activity through entrainment of single units and to display high spectral coherence with PFC LFPs, unit firing and ensemble/population behavior depending on task demands (Hyman et al., 2005; Jones and Wilson, 2005; Siapas et al., 2005; Sirota et al., 2008; Benchenane et al., 2010). For instance, Hyman et al. (2010) reported that 94% of theta-modulated mPFC neurons displayed significantly greater phase-locking to hippocampal theta during correct trials of a delayed non-match-toposition task, than during error trials. Darling et al. (2011) used theta-contingent trace EBC to assess the role of hippocampal pretrial theta state on neural response patterns in the caudal anterior cingulate subregion of mPFC (Figure 6), which has been shown to be critical for normal acquisition of trace EBC (Kronforst-Collins and Disterhoft, 1998; Weible et al., 2000). Multi-unit responses in T+ animals showed an inhibitory/excitatory (I/E) response pattern to the tone that was not present in T− animals or unpaired controls. These T+ units also showed increased and persistent excitation during the trace and US periods. The I/E sequence was consistent with a previous study by Weible et al. (2003) that found single-units in the caudal anterior cingulate cortex that show inhibition followed by excitation in response to the tone followed by excitation through the trace and US periods. A different population of cells did not display the I/E sequence and failed to respond during the trace interval. The authors proposed that the I/E sequence provides a signal-to-noise enhancement that increases the salience of the tone. One functionally important outcome would be persistent unit firing from PFC to the lateral pontine nucleus, bridging the gap in the trace period that cannot be accommodated by brainstem or cerebellar circuits alone. Darling et al. (2011) study, which recorded from the more caudal portion (1.0 mm anterior to bregma) of the Weible et al. range (~0–5.0 mm anterior to bregma), showed that these two distinct populations of cells were specific to hippocampal theta state. Interestingly, mPFC LFPs did not oscillate at theta frequency, suggesting that the two unit response profiles were modulated by the presence or absence of theta in the hippocampus. This may indicate that differences in hippocampal pretrial theta state are reflected in the response patterns of hippocampal output neurons projecting to relevant structures during the EBC paradigm. This finding is critical for future analyses of theta as it demonstrates that behaviorally significant information about theta state and phase can be transmitted to target structures even if the theta band LFP is not present in those structures.

**Figure 7:**
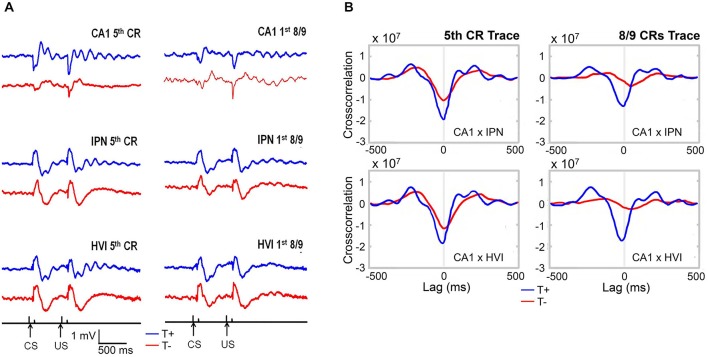
**(A)** Average hippocampal and cerebellar LFPs during early (5th CR) and late (8/9 CRs) theta-contingent trace eyeblink learning. T+ LFPs (blue) display consistent short-latency onset of robust and rhythmic theta oscillations that are precisely time-locked to the CS and US occurrences. T− LFPs (red) are notably less organized at theta frequency across trials and days. **(B)** Hippocampo-cerebellar crosscorrelograms during the stimulus-free trace interval for early and late theta-contingent trace eyeblink learning show rhythmic, anti-phasic, synchronization of hippocampus and both cerebellar regions at theta frequency during T+ (blue) and not T− (red) trace periods.

In light of these extra-hippocampal effects, Hoffmann and Berry (2009) sought to identify influence of hippocampal theta-contingent training on cerebellar circuitry known to be essential to all forms of EBC. Recording simultaneously from CA1, IPN, and cerebellar cortical lobule HVI during trace conditioning (Figure 7), they replicated the behavioral benefits of T+ training and found several functionally relevant electrophysiological phenomena in T+ animals including: (1) modulation of cerebellar evoked response amplitude, (2) cerebellar theta oscillations time-locked to the conditioning stimuli, (3) precise (180 degree) phase synchronization of hippocampus and cerebellar IPN and HVI LFPs at theta frequency; and (4) theta frequency phase synchronization (0 degree) between IPN and HVI. Prior to this experiment, inactivation studies had suggested that the hippocampus and cerebellum interact in important ways during the task but it was unclear how this was accomplished since no direct pathways between the two structures have been identified or directly manipulated in the rabbit. Our theta-contingent training technology allowed hippocampal theta to be used as a quasi-independent variable to further address this question. The resultant findings of very strong and precise T+ rhythmic coordination of hippocampal and cerebellar LFPs suggested hippocampal theta as a means for establishing a flexible long-distance functional connection between these two structures and perhaps as a system-wide means for regulating the functional properties of the anatomically distributed circuit for trace EBC. The precise phase locking over the substantial distance between hippocampus and cerebellum would be surprising if one region were directly driving the other and, instead, suggests a common pacemaker for both structures. If so, it is likely not a unitary pacemaker, as we have observed theta presence in either hippocampus or cerebellum while it is not present in the other. Regardless of the source(s) of this hippocampal–cerebellar synchrony, it can be concluded that a brain state indexed by hippocampal theta (and here exploited by our BCI) serves as a reliable predictor of rhythmic vs. non-rhythmic modes of information processing in the cerebellum.

At present, the origins and pacers of theta within the hippocampus are well documented, however, it is not well established what extra-cerebellar inputs and internal mechanisms might mediate theta coordination between hippocampus and cerebellum. It has been shown that low frequency theta is a strong organizing principle in the cerebellum. Golgi and other cells of the cerebellar granule cell layer have been widely reported to have intrinsic resonant properties at theta frequency, which would serve to amplify any theta field oscillation in the structure (D’Angelo et al., 2001; Dugué et al., 2009; Mathy et al., 2009; Gandolfi et al., 2013). However, there are a number of input structures that could serve as candidates for any potential pacing or entrainment function during hippocampo-cerebellar synchronization. Based on anatomical connectivity and relevance to theta pacing, some of the most likely candidates include pontine nuclei as a component of the reticular activating system and the supramammillary and mammillary nuclei of the hypothalamus. Though inferior olive is likely involved as a theta-supporting input structure for cerebellum (Marshall and Lang, 2004), lack of a clear route to telencephalon make it a less likely candidate for bidirectional forebrain-cerebellum synchronization.

Though the MS-vDBB has been established as the major pacer of hippocampal theta activity, dynamic interplay between MS-vDBB and supramammillary nucleus has been reported to provide transient reversal of theta driving influences (Kocsis, 2006). These findings provide a concrete example of a system in which hippocampus and cerebellum (which receives direct projections from supramammillary nucleus) could flexibly synchronize. In extending this possible mechanism to the temporally-specific coordination differences in the context of the above results, it may be interesting to explore this dynamic MS-supramammillary nucleus directionality as a potential mediator of shifts in global synchronization by CA1 or IPN theta-triggered conditions in early and late learning, respectively. It is possible that shifts in EBC circuitry demands could influence the direction of theta pacing within MS-supramammillary connections in ways that could better synchronize in one direction than the other at certain stages of learning. Alternatively, because both hippocampus and cerebellum have internal resonance properties at theta and because external stimuli could serve an external resetting function for oscillation that would time lock the LFPs, it is possible that more than one pathway or type of input (rather than a unitary source/system) mediates theta synchronization of hippocampus and cerebellum. MS pacemaking neurons act as relaxation oscillators, which can serve to reset hippocampal LFPs that behave as harmonic oscillators (Buzsáki, 2006). Perhaps, as a function of context, expectancy or other state, MS neurons exhibiting theta pacemaking properties prior to tone onset could operate in ways that would allow external stimuli to reset the oscillations more quickly in each brain region and with a precise phase relationship (as opposed to T− which might have to transition from delta to theta in response to stimuli and therefore the oscillator would appear to “wobble” or take several cycles to establish a stable, coherent frequency). For this synchronization scheme we must consider the roles that additional EBC structures, such as mPFC, lateral pontine nucleus and inferior olive, might play along with the well-established septo-hippocampal/supramammillary theta systems. These EBC regions are important to the pathways processing CS and US information and could provide resetting input to cerebellum. Hippocampal learning-related activity and theta are known to modulate PFC activity (Mehta et al., 2002; Hyman et al., 2005; Jones and Wilson, 2005; Siapas et al., 2005; Darling et al., 2011). Persistent PFC CS-related input is transmitted to cerebellum via lateral pontine mossy and parallel fibers during trace EBC learning so that CS information can overlap with temporally-separated US information (Kalmbach et al., 2009; see Figure 1B for circuit diagram). This PFC/pontine-mediated CS-US convergence is critical for trace EBC. However, it is unknown if this input to the pons can be modulated by theta in ways that might provide another directional influence for forebrain-cerebellar theta synchronization that over the course of training. Furthermore, evidence that US-related activity is transmitted via climbing fibers from inferior olive to cerebellum, and that inferior olive supports theta frequency oscillations, evoke the possibility that olivary input about the US might be theta modulated in ways that could modify the learning process. The inferior olive is a major component of the error correction model for EBC: inferior olive is actively inhibited by IPN when CRs are performed correctly (and is active when CR is performed incorrectly). In such a case, inferior olive could help to synchronize the cerebellum at theta frequency for US-related responses or during certain phases relevant to the error correction model (either late in learning when CRs are correctly emitted (and IPN is actively inhibiting inferior olive) or early in learning when they are less likely (and IPN is not inhibiting inferior olive).

Perhaps one of the most widely suggested pathways for hippocampal-cerebellar interactions (and perhaps theta modulation of EBC circuitry) is via PFC and lateral pontine nucleus (for example Weiss and Disterhoft, 2011). General forebrain-cerebellar interactions measured in unit activity during EBC have been extensively reported (e.g., Green and Arenos, 2007; Siegel et al., 2012). Kalmbach et al. (2009) have reported on prefrontal-cerebellar interactions during hippocampal-dependent trace EBC. These authors demonstrated prelimbic/infralimbic modulation of cerebellar responses. Interactions between the hippocampus and PFC are also important to consider in this potentially theta modulated pathway. Direct connections between these two regions involve projections from the ventral hippocampus, which is less involved in EBC than the dorsal hippocampus (Weible et al., 2000). An alternative explanation for hippocampal-prefrontal interactions put forth recently by Hasselmo and Sarter (2011) might be that both structures receive common cholinergic facilitation during conditioning, as a result of which the two circuits could work together in parallel rather than in a serial arrangement. This notion is buttressed by the fact that hippocampal theta is known to entrain forebrain areas during learning- and memory-related tasks (e.g., Hyman et al., 2005; Siapas et al., 2005; Darling et al., 2011). To the extent that mPFC receives learning-related input from hippocampus about CS-US associations and that hippocampal oscillatory activity entrains mPFC response patterns, the findings put forth by Kalmbach and others from the Mauk lab provides tentative support for hippocampal influence on cerebellar physiology and a viable route for exerting such influence. As mentioned earlier, a direct anatomical link between hippocampus and cerebellum has been documented in a variety of species (Heath and Harper, 1974; Snider and Maiti, 1976; Newman and Reza, 1979; Liu et al., 2012; Arrigo et al., 2014). Though none have yet been identified in the rabbit, this pathway remains a possibility.

Although rhythmic multi- (Kirk, 1997) and single-unit (Kirk et al., 1996) activity at theta frequency in the supramammillary nucleus and posterior hypothalamic nucleus have been shown to survive septal infusions of procaine (which abolished hippocampal theta), phasic cell discharge in the medial mammillary nucleus does not. This suggests that the medial mammillary nucleus (dependent on hippocampal, entorhinal cortex and MS inputs) is a downstream target for septally-driven hippocampal theta while, posterior hypothalamic and supramammillary nuclei provide mostly ascending phasic input to MS. Despite the lack of a theta-modulated effect on supramammillary nucleus in Kirk’s reports, Kocsis (2006) has provided evidence of bidirectional coupling between MS and supramammillary theta generators. This report indicated that septohippocampal theta can precede supramammillary phase-locked firing. These instances of descending influence on supramammillary nucleus were related to slow, spontaneously generated theta onset, while instances of ascending influences of supramammillary nucleus on MS phase-locked unit firing were related to accelerations in theta frequency during sensory or brainstem stimulation.

Building on these findings, a number of authors have reported a variety of direct projections from hypothalamic nuclei, including the medial mammillary and supramammillary nuclei, to cerebellar cortical regions or IPN or both (e.g., Dietrichs and Haines, 1989; Vertes, 1992; Dietrichs et al., 1994; for summary of relevant anatomical connections, see Figure 3). Retrograde labeling shows that the caudal half of hypothalamus projects to more lateral cerebellar nuclei (dentate, anterior and posterior interpositus; Dietrichs and Haines, 1989). Additionally, a number of indirect pathways from medial mammillary and supramammillary nuclei to cerebellum exist, including strong projections to relays in inferior olive and pontine nuclei (Dietrichs and Walberg, 1981; Aas and Brodal, 1988, 1989; Dietrichs et al., 1994). Hypothalamo-ponto-cerebellar pathways are known to terminate in cerebellar cortex only (not nuclei; Aas and Brodal, 1988, 1989). Medial mammillary nucleus, specifically, is known to project heavily to the anterior thalamic nucleus, which in turn has been shown to relay theta-frequency activity back to the hippocampus via entorhinal cortex and cingulate cortex (Aas and Brodal, 1988, 1989), as well as to the pontine nuclei (and on to cerebellum; Dietrichs and Haines, 1989). Since the majority of areas noted in both direct and indirect pathways have been shown to reflect theta modulated activity patterns, it is possible that these subcortical connections could provide an alternative pathway for hippocampal-cerebellar theta synchronization. Specifically, the possibility remains that medial mammillary nucleus may act as a gateway structure for distributed downstream targets to receive theta related inputs.

Additionally, the MS-vDBB projects to a large number of brain regions that show theta modulation, including all hippocampal sub-regions entorhinal cortex, perirhinal and retrosplenial cortices, PFC, medial mammillary and supramammillary nuclei, anterior nuclei of the thalamus, amygdala, inferior colliculus, and several brainstem nuclei (Kirk et al., 1996; Kirk, 1998; Hyman et al., 2005; Siapas et al., 2005; Kocsis, 2006; Tsanov et al., 2011). Thus, a number of possibilities for a unitary pacemaker for hippocampus and other regions are available. However, interactions between hippocampus or MS and cerebellar regions are likely polysynaptic and mediated by at least one additional structure.

## Benefit of Theta-Contingent Training in Aged Animals

**Figure 8:**
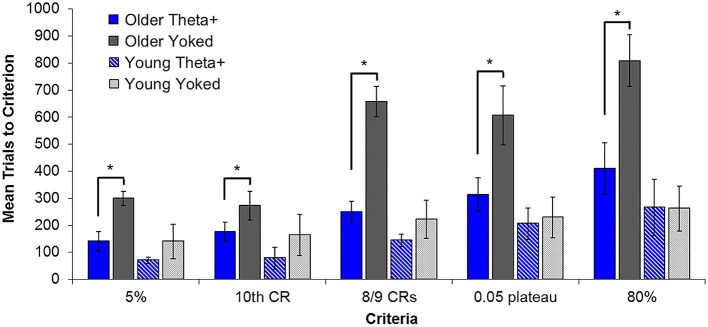
**Average number of training trials required by each group to attain classic behavioral learning criteria and those criteria defined by the state-space model**. Older T+ animals reached all learning criteria significantly faster than their yoked controls, while young T+ animals were not significantly different from their yoked counterparts on any criteria except 8/9 CRs. When behavioral criteria were organized according to early and later learning phases (5% and 10th CR vs. 8/9 CRs, 0.05 plateau and 80%), there was a highly significant interaction of age, treatment, and phase. *Post hoc* comparisons substantiated a significant benefit of theta triggering in the early phase for both young and older groups. In contrast, the later learning phase showed no theta-triggering benefit in young animals, but a continuing benefit in the older group. Asterisks indicate *p* < 0.05.

To address the role of theta in age-related memory performance, Asaka et al. (2005) extended this interface-mediated enhancement to include trace EBC in aging rabbits. Young (~5 months) and aged (~28 months) rabbits were trained during theta (T+) or as yoked controls with identical inter-trial intervals, with hippocampal theta state uncontrolled. Aged animals in the T+ condition were faster to reach an early learning criterion (10th CR) and later criteria (8/9 CRs and 80% CRs) than yoked controls (Figure 8). The learning rates of young T+ animals were facilitated early in conditioning, however T+ training did not enhance learning rate for later criteria. These results suggest that the aging deficit in EBC may include theta-related performance impairments as well as a delay in acquiring the initial association between conditioning stimuli. This behavioral performance aspect of theta becomes important in understanding phases of learning and, perhaps, in understanding the relationship between hippocampus and cerebellum during the asymptotic stages of learning. In addition, this study compared traditional early and asymptotic learning criteria to learning trajectories characterized by a state-space learning model developed by Smith et al. (2004). The state-space model used Bernoulli probability to describe the observed binary-valued behavioral responses (CR or UR) and a Gaussian state equation (random walk model) that served to describe the unobservable learning state process. This state-space model defined learning curves for individual animals as the probability of a correct response as a function of the learning state process. For each animal, a 5% baseline criterion, which estimated the end of learning phase 1, and a 0.05 plateau criterion estimating the onset of asymptotic responding were identified. The 5% baseline criterion was represented as the training trial on which the ideal observer was 95% certain that the animal would emit CRs at a rate above chance (5%) for the rest of the experiment. The 0.05 plateau criterion was defined as the point, after passing the 5% criterion, at which the change in the probability of a correct response was less than 5% for 10 consecutive trials. Using these criteria, the model was able to identify the precise “learning trial”—the first trial on which CR performance will be, with reasonable certainty, better than chance for the rest of training on a subject-by-subject basis. These indices replicated the significant behavioral effects of theta-contingent training and provided theoretical and mathematical support for the use of traditional learning criteria. It should be emphasized that older animals trained in the T+ condition learned as quickly as the young yoked controls, thereby ameliorating age-related learning impairment non-pharmacologically to, for example, enhance cholinergic function. Since the integrity of 3–7 Hz theta is dependent on cholinergic activity, this study provides support for the claim that even an aging, cholingerically-impaired brain may exhibit useful periods of relatively normal theta during which learning may be unimpaired.

## Cholinergic Modulation of Cognitive Function

Among other neurotransmitters, neurotropic factors, intracellular molecular cascades and transcription factors, there is considerable evidence to suggest that central cholinergic systems may be important neural substrates for cognitive functions such as attention and memory encoding (Robbins et al., 1989). Cholinergic neurons are widely distributed throughout the mammalian brain and exist as both projection neurons and interneurons. Among the most prominent populations of cholinergic projection neurons are those located in the basal forebrain (MS, vertical and horizontal limbs of the DBB, and the nucleus basalis/substantia inominata) and those located in the laterodorsal and pedunculopontine tegmental nuclei of the brainstem. While brainstem cholinergic neurons primarily innervate regions of the thalamus and basal ganglia, a number of anatomically distinct subpopulations have been identified in basal forebrain: Cholinergic neurons of the medial septum/vertical limb predominantly project to the dorsal hippocampus and dentate gyrus, while those found in the horizontal limb and nucleus basalis primarily innervate distinct areas of the cortical mantle, including mPFC (Mesulam et al., 1983a,b; Chandler et al., 2013). Such anatomical distinctions are accompanied by phenotypic and co-transmitter differences and distinct functional relevance for each subpopulation (Melander et al., 1985; Geula et al., 1993). A recent model proposes diverse functional roles for basal forebrain cholinergic projections to the PFC and hippocampus (Hasselmo and Sarter, 2011). Within this model, cholinergic modulation of target regions supports cue detection and attentional performance (in PFC) as well as encoding of information into long-term memory (in hippocampus). It is well known that 3–7 Hz hippocampal theta relies on functional cholinergic systems, since administration of anticholinergic drugs to the MS disrupts the regularity and amplitude of theta waves (Solomon and Gottfried, 1981; Asaka et al., 2002). As such, our theta-contingent trial presentation provides a means to study cognitive function under pro-cholinergic conditions (T+) and periods when the cholinergic system (MS/basal forebrain) is not functioning strongly (T−). Behaviorally, as previously discussed, our theta-contingent training has been used to alleviate age-related learning impairments that have commonly been attributed to disruptions in the cholinergic system (Asaka et al., 2002).

Cholinergic modulation has been shown by lesions of cholinergic forebrain projections to the cortex to be necessary for performance of tasks requiring sustained attentional processes (Chiba et al., 1995; McGaughy et al., 1996; Baxter et al., 1999; Newman and McGaughy, 2008). These tasks require the subject to attend to a transient cue stimulus over the course of a session and discriminate between signaled and non-signaled events. Forebrain lesions have failed to disrupt tasks such as the radial arm maze (Vuckovich et al., 2004) and T-maze alternation (Pang and Nocera, 1999) that do not explicitly tax attention due to the presence of multiple static cues available for navigation rather than forcing the subject to attend to a subset of available cues. Furthermore, measurement of ACh release in the mPFC during a cued task revealed a relationship between ACh release and cue detection (Parikh et al., 2007). Temporary ACh level increases were seen in the mPFC only on trials in which the cue was detected. These results provide support for the notion that cholinergic modulation enhances cue detection, possibly by enhancing the salience of the cue. Several studies have shown that ACh receptor activation serves to enhance the firing of subsets of neurons in the cortex (Krnjević et al., 1971; Hsieh et al., 2000; Gulledge et al., 2009) while simultaneously reducing the spread of neural activity at excitatory recurrent synapses (Hasselmo and Bower, 1992; Hasselmo and Cekic, 1996). This inhibition of background firing and enhancement of sensory-evoked responding is comparable to the CS-evoked inhibitory/excitatory (I/E) sequence seen in mPFC units in our T+ animals (Darling et al., 2011). The I/E sequence has been interpreted as a signal-to-noise enhancement that increases the salience of the tone (Weible et al., 2003; Darling et al., 2011) as would be expected in a pro-cholinergic (T+) state based on the model.

Furthermore, our theta-triggered results agree with the general idea that the dynamics of the local circuits likely contribute to the effect of cholinergic modulation in the targeted area. As expected based on the model, the pro-cholinergic state (T+) resulted in enhancement of cellular activity specific to the functional area, such as increased unit firing late in the trace period in the hippocampus (Griffin et al., 2004; Darling et al., 2011) and a signal-to-noise enhancement with persistent activity through the trace in the mPFC (Darling et al., 2011). Additionally, our findings indicate that the pro-cholinergic conditions reflected by our T+ training may also affect circuit dynamics in the cerebellum, as seen in the LFP responses (Hoffmann and Berry, 2009). Importantly, our BCI can also be used to examine the cellular responding under conditions (T−) when the cholinergic system is not engaged, providing a model for cognitive dysfunctions without the use of drugs or lesions that may produce undesirable side effects.

## Separate Encoding and Retrieval Theta Phase Model

The ability of our BCI to engage naturally occurring theta may serve to coordinate activity both within the hippocampus at the cellular level and across other structures necessary for learning the EBC paradigm. This general idea is consistent with several theories and models of the role of neurobiological oscillations in learning (Jensen, 2001; Buzsáki, 2002; Hasselmo et al., 2002; Buzsáki and Draguhn, 2004; Cutsuridis et al., 2010; Lisman and Jensen, 2013). One prominent model has suggested that the hippocampal theta rhythm provides separate encoding (peak of CA1 theta) and retrieval (trough of CA1 theta) phases, allowing for strengthening or weakening (plasticity) or readout from memory (stability), respectively (Hasselmo et al., 2002). This theory has received empirical support in studies showing LTP induction at theta peaks and LTD induction at theta troughs following stimulation in hippocampal slice preparations (Huerta and Lisman, 1995), anesthetized animals (Holscher et al., 1997), and behaving animals (Hyman et al., 2003). These alternating phases are possible due to fluctuations in output strength from the entorhinal cortex and CA3 to CA1. They have proposed that the encoding phase is characterized by strong input from entorhinal cortex with weak input from CA3. Conversely, during the retrieval phase synaptic input from CA3 is strong and entorhinal input is weak, as well as stronger output from CA1. This model has been expanded upon to include single-unit firing of pyramidal cells and several classes of interneurons in CA1 (Cutsuridis et al., 2010). Using established theta phase preferences, the authors provide a cellular mechanism through which CA1 is able to alternate between these periods of encoding and retrieval.

**Figure 9:**
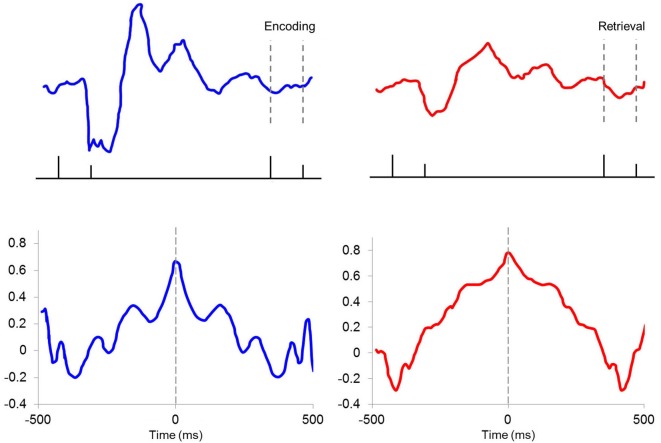
**Encoding and retrieval phases for T+ and T− LFPs.** Average LFPs for T+ (top left) and T− (top right) animals showing (ticks from left to right) tone onset, tone offset, airpuff onset, and airpuff offset. Differences in theta rhythm reset results in T+ animals receiving the US during a theta peak, while T− animals receive the US during a theta trough, corresponding to encoding and retrieval phases of the Hasselmo model, respectively. Autocorrelations of multi-unit responding during the trace period show rhythmicity in T+ units (bottom left) that is not seen in T− units (bottom right).

In this context, we can identify a potential mechanism underlying the behavioral benefits associated with theta-contingent training. The aforementioned theta phase reset following CS presentation (Hoffmann and Berry, 2009; Darling et al., 2011; Figures 7, 9) allows animals in the T+ condition to consistently receive the US during a peak of theta (measured at the pyramidal cell layer). This promotes development of the CS-US association in T+ animals as the US is present during an encoding phase, while T− animals experience a less coherent phase reset and, consequently, inconsistent delivery of the US relative to the theta rhythm. Importantly, with the US arriving during an encoding phase of theta, the hippocampus is consistently in a retrieval mode shortly before airpuff presentation when adaptive CRs are expected. At the cellular level, our multi-unit recordings, likely dominated by pyramidal cell activity, show increased excitation in the T+ condition late in the trace period. This is consistent with the notion of increased CA1 output to entorhinal cortex during retrieval (Griffin et al., 2004; Darling, 2008; Darling et al., 2011; Figures 4, 5). While Darling’s study collapsed several interneuron subtypes into one general group, our more recent data characterize the response profiles of several classes of GABAergic interneurons under theta and non-theta conditions during EBC. These findings are in line with the Cutsuridis model—patterns of interneuron firing that are consistent with rapid acquisition tending to occur during theta, whereas firing patterns favorable to memory retrieval appear to disrupt early stages of learning in our T− group (Cicchese, 2013). Such results lend important empirical support for the model in behaving animals. Accompanying the differences in overt performance between T+ and T−, behaviorally significant events (stimuli) and their LFP responses coincide with the model’s proposed phases of encoding (US arrival) and retrieval (time of adaptive CRs and increased hippocampal output) under T+, but not T−, conditioning (Griffin et al., 2004; Darling, 2008; Darling et al., 2011). This provides empirical support for a possible behavioral role of theta phases within an established model of associative learning.

## Concluding Remarks

Evidence from empirical research and theoretical models suggest that theta oscillations provide the basis for wide-ranging coordination of distributed brain systems, with cellular responses (including plasticity) resonating to inputs that are related to theta phase (Buzsáki, 2002, 2006; Hasselmo et al., 2002; Hasselmo, 2005). Our methods provide a powerful tool for controlling and analyzing theta-based temporal coherence throughout the EBC system and the neural mechanisms that underlie the observed cognitive-behavioral benefits. The reviewed findings on the role of hippocampal oscillatory potentials in cognitive processes throughout the brain, raise important questions on the nature and impact of precise theta coordination in distributed brain systems and the potential benefit of manipulating theta to optimize learning. Conversely, our interface-controlled non-theta state may be a drug-free, non-lesion model of psychiatric/cognitive disorders such as schizophrenia, autism and age-related memory impairments, thought to result from desynchronization of critical brain systems (Behrendt and Young, 2004; Spencer et al., 2004; Asaka et al., 2005; Donkers et al., 2011; Doesburg et al., 2013). Continuation of this approach will enhance our knowledge of the roles of LFPs as well as their relation to signals in other bandwidths and time frames (Bullock, 1997), with the goal of a comprehensive description of electrophysiological mechanisms of information processing.

## Conflict of Interest Statement

The authors declare that the research was conducted in the absence of any commercial or financial relationships that could be construed as a potential conflict of interest.

## Abbreviations

ACh: acetylcholine
BCI: brain-computer interface
CA1–CA3: Cornu Ammonis subdivisions 1–3
CR: conditioned response
CS: conditioned stimulus
EBC: eyeblink classical conditioning
HVI: Larsell’s hemispheric lobule VI
I/E: inhibitory/excitatory
IPN: interpositus nucleus
LFP: local field potential
LTD: long-term depression
LTP: long-term potentiation
mPFC: medial prefrontal cortex
MS: medial septum
T+: theta-triggered
T−: non-theta-triggered
US: unconditioned stimulus
vDBB: vertical limb of the diagonal band of Broca.
